# Save or Remove the Accessory Spleen in Patient Candidates to Splenectomy for Lymphoproliferative Disorders: Rational and Literature Review

**DOI:** 10.1002/ccr3.70735

**Published:** 2025-09-22

**Authors:** Valerio Rinaldi, Michela Ribersani, Carla Giordano, Francesca Maccioni, Iulia Catalina Ferent, Priscilla Nardi, Roberto Caronna, Paolina Saullo

**Affiliations:** ^1^ Department of Surgery Sapienza University of Rome Rome Italy; ^2^ Department of Traslational and Precision Medicine Sapienza University of Rome Rome Italy; ^3^ Department of Radiological, Oncological and Pathological Sciences Sapienza University of Rome Rome Italy

**Keywords:** accessory spleen, case reports, lymphoma, lymphoproliferative disorders, splenectomy

## Abstract

Sparing or removing the accessory spleen (AS) in patients undergoing diagnostic splenectomy for lymphoproliferative disorders (LPD) is an unsolved issue rarely considered. Usually, a healthy AS can be preserved. Reporting a case of our own, this paper aims to review existing literature and propose a tailored approach. A 59‐year‐old woman presented with unexplained anemia. A FDG‐PET‐CT scan revealed splenomegaly and two hypermetabolic splenic nodules, pathological upper and lower diaphragmatic lymph nodes, and a non‐FDG‐avid AS around splenic hilum. The patient underwent diagnostic splenectomy and AS removal. The histopathological examination of the spleen disclosed a diffuse large B‐cell lymphoma (DLBCL), whereas the AS was uninvolved, consistent with the preoperative FDG‐PET‐CT findings. The post‐operative course was uneventful. The patient was discharged on the 5th post‐operative day. Asplenia following splenectomy increases the risk of severe infections. It is hypothesized that sparing a healthy AS could help maintain immune competence. However, there is currently insufficient evidence to guide the decision on whether to remove or spare an AS during splenectomy for diagnostic purposes in suspected LPD cases, and to understand the potential prognostic impact (both oncological and infectious) of this strategy. The preoperative finding of a non‐FDG‐avid AS may recommend surgical sparing.

**Summary**

- It is unclear whether to remove or preserve AS during splenectomy for LPD, and the oncological or infectious impact is uncertain.
- Preoperative FDG‐PET can guide the decision: if FDG‐PET is negative, AS can be preserved; if positive, other eventual disease sites, if present, should influence the choice.

## 1 Introduction

An accessory spleen (AS) is a small mass of splenic tissue detached from the main spleen, located in the abdomen. It is also described as “supernumerary spleen” or “splenunculus” [1]. The presence of an AS is associated with congenital abnormalities in the development of the dorsal mesogastrium during embryonic life [2]. AS may occur as single or multiple entities. In contrast, splenic tissue implants can disseminate within the peritoneal cavity following traumatic spleen rupture, a condition known as splenosis [3]. However, splenosis should not be confused with congenital AS and will not be addressed in this study. AS is most commonly found near the splenic hilum (80% of cases), pancreatic tail (20%), or in proximity to the stomach, and it is almost always asymptomatic. Typically, AS measures 1–3 cm in size [4], and its detection has increased because of improvements in diagnostic imaging. AS could be misdiagnosed as enlarged lymph nodes of the splenic hilum, gastroenteropancreatic neuroendocrine tumors (GEP‐NETs) or lymphomatous disease [5]. We present the case of a 59‐year‐old woman with splenomegaly and suspected splenic lymphoproliferative disease. A small AS was detected near the splenic hilum. Diagnostic splenectomy along with AS removal was performed because of the absence of a conclusive histopathological diagnosis. The question of whether the AS should be spared was raised postoperatively. We conducted a literature review to assess whether *sparing* the AS may reduce post‐splenectomy infectious complications, whether it may pose a risk for recurrence of lymphoproliferative disease (LPD), and whether preoperative FDG‐PET‐CT scans can reliably assess lymphomatous involvement of the AS. The clinical case was managed in an academic setting, and the case report was structured following the SCARE guidelines [6].

## 2 Patient Information

Our study describes a 59‐year‐old Caucasian woman with no significant medical history. She was referred to the Hematology Department for evaluation of anemia.

Her past medical and surgical history was unremarkable, with no comorbidities. She was not on any medications and was a former smoker. There was no family history of relevant diseases.

## 3 Informed Consent and Ethical Approval

Written informed consent was obtained from the patient to publish this case report. Ethical review and approval were waived for this study because of its retrospective nature.

## 4 Clinical Findings

On physical examination, the spleen was palpable in the left hypochondrium. No palpable lymph nodes were detected in any of the superficial lymph node stations.

## 5 Timeline

The patient was initially evaluated by hematologists in September 2022 for anemia of unknown origin. In November 2022, a total body CT scan was performed, raising the suspicion of splenic lymphoma. Consequently, a peripheral blood smear and bone marrow biopsy were conducted. In December 2022, an FDG‐PET‐CT scan was carried out, followed by a skin biopsy for a suspicious FDG‐PET‐CT‐positive skin lesion. In January 2023, the patient underwent an open splenectomy, along with AS removal.

## 6 Diagnostic Assessment

**FIGURE 1:**
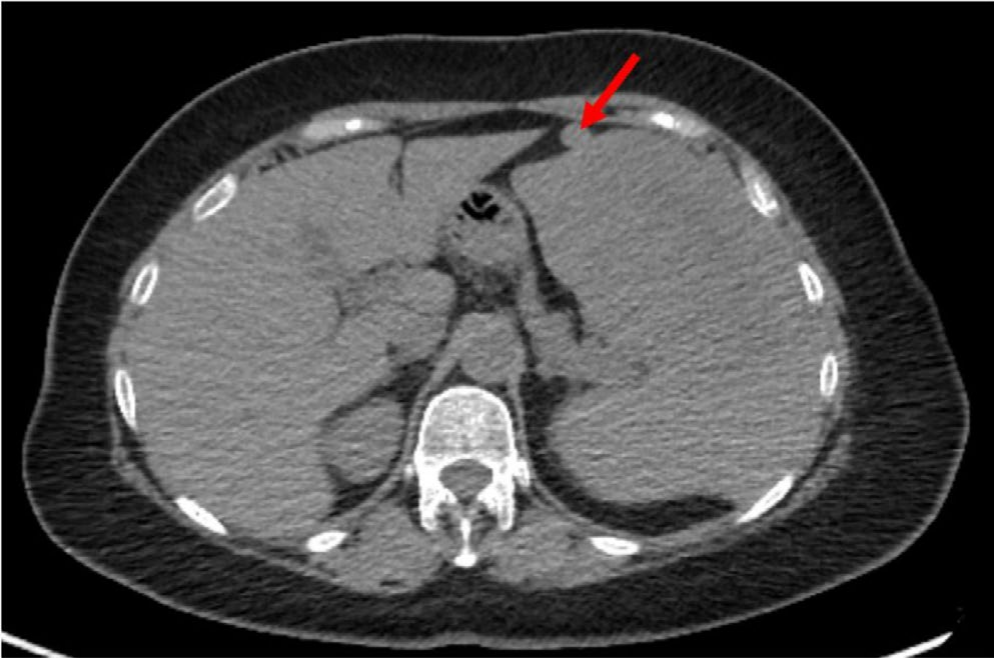
Splenomegaly and accessory spleen (red arrow).

**FIGURE 2:**
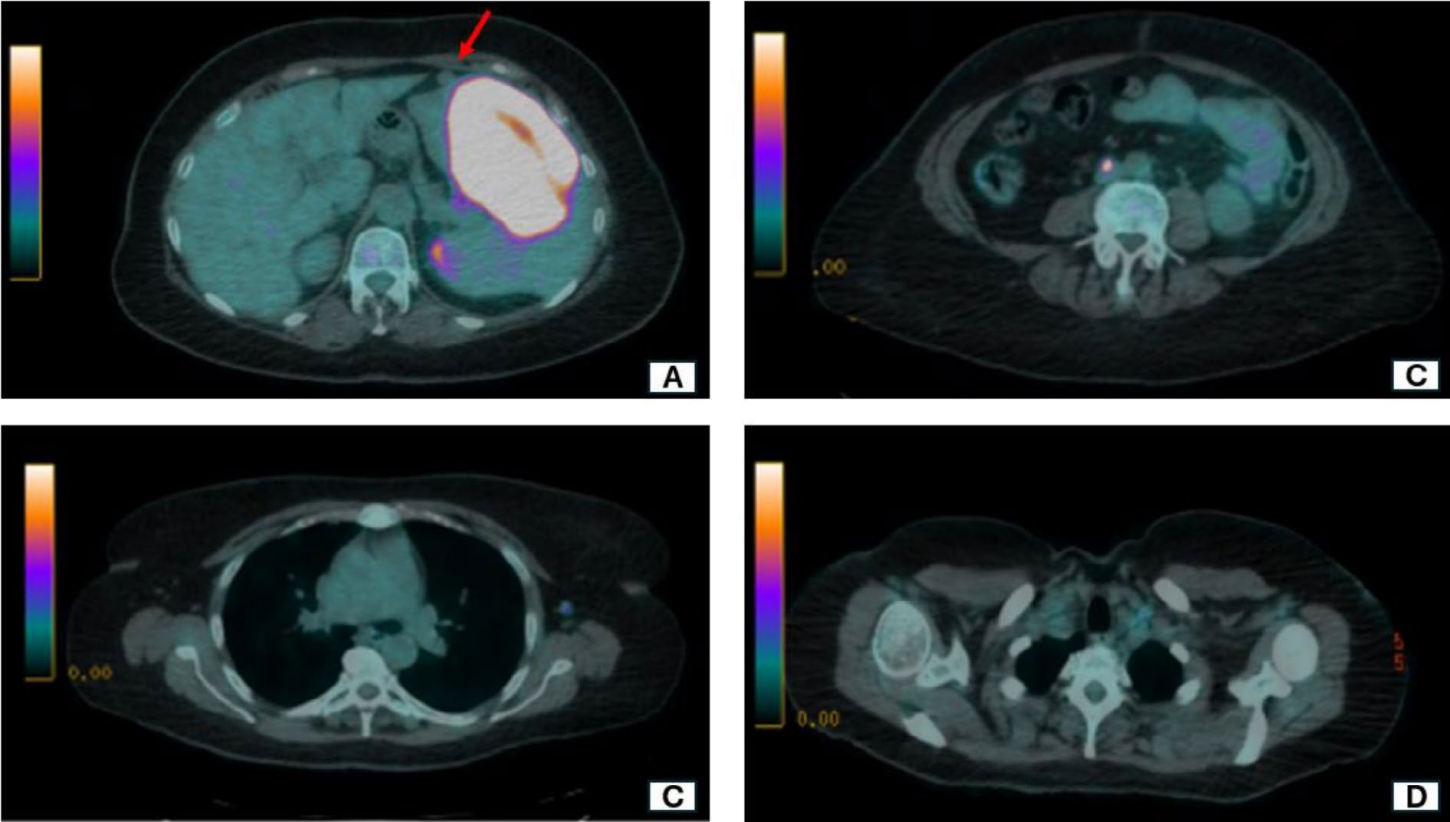
(A) Hypermetabolic splenic nodules and no uptake shown by the accessory spleen (red arrow). (B) Lower diaphragmatic lymph nodes. (C, D) Upper diaphragmatic lymph nodes.

A Total Body CT scan revealed splenomegaly (maximum diameter: 17 cm) with two splenic nodules measuring 12 and 4 cm, respectively. The liver appeared normal in both morphology and size. Additionally, the CT scan identified an accessory spleen (AS) with a maximum diameter of 3 cm, located on the anterior edge of the spleen (Figure 1). Suspecting lymphoproliferative disease (LPD), both a peripheral blood smear and a bone marrow biopsy were performed, yielding negative results. An FDG‐PET‐CT scan revealed high uptake in the two splenic nodules (SUV max 28.8) with moderate uptake in the upper and lower diaphragmatic lymph nodes (para‐aortic: SUV max 4.1; para‐caval: SUV max 7.3; left common iliac: SUV max 4.2) and in an anterolateral thoracic wall subcutaneous lesion (SUV max 9.2) (Figure 2). No FDG uptake was observed in the AS and liver. The subcutaneous lesion was excised, and the histopathological analysis suggested a B‐cell lymphoproliferative disease (CD20+, CD3‐, CD5‐, and CD23‐ lymphocytes), without a firm diagnosis. The hematology board recommended splenectomy to obtain a definitive diagnosis rather than fine‐needle aspiration (FNA) or core needle biopsy (CNB) because of post‐procedural complications recently observed at our institution, which led to significant delays in hematological treatment and unfavorable prognostic outcomes. Consequently, the patient was referred for surgery to complete the diagnostic process.

On the basis of clinical and radiological evidence, splenectomy was scheduled to obtain a conclusive diagnosis of the main localization of LPD.

## 7 Therapeutic Interventions

No specific preoperative measures were required except for fasting, which began the night before the surgery.

**FIGURE 3:**
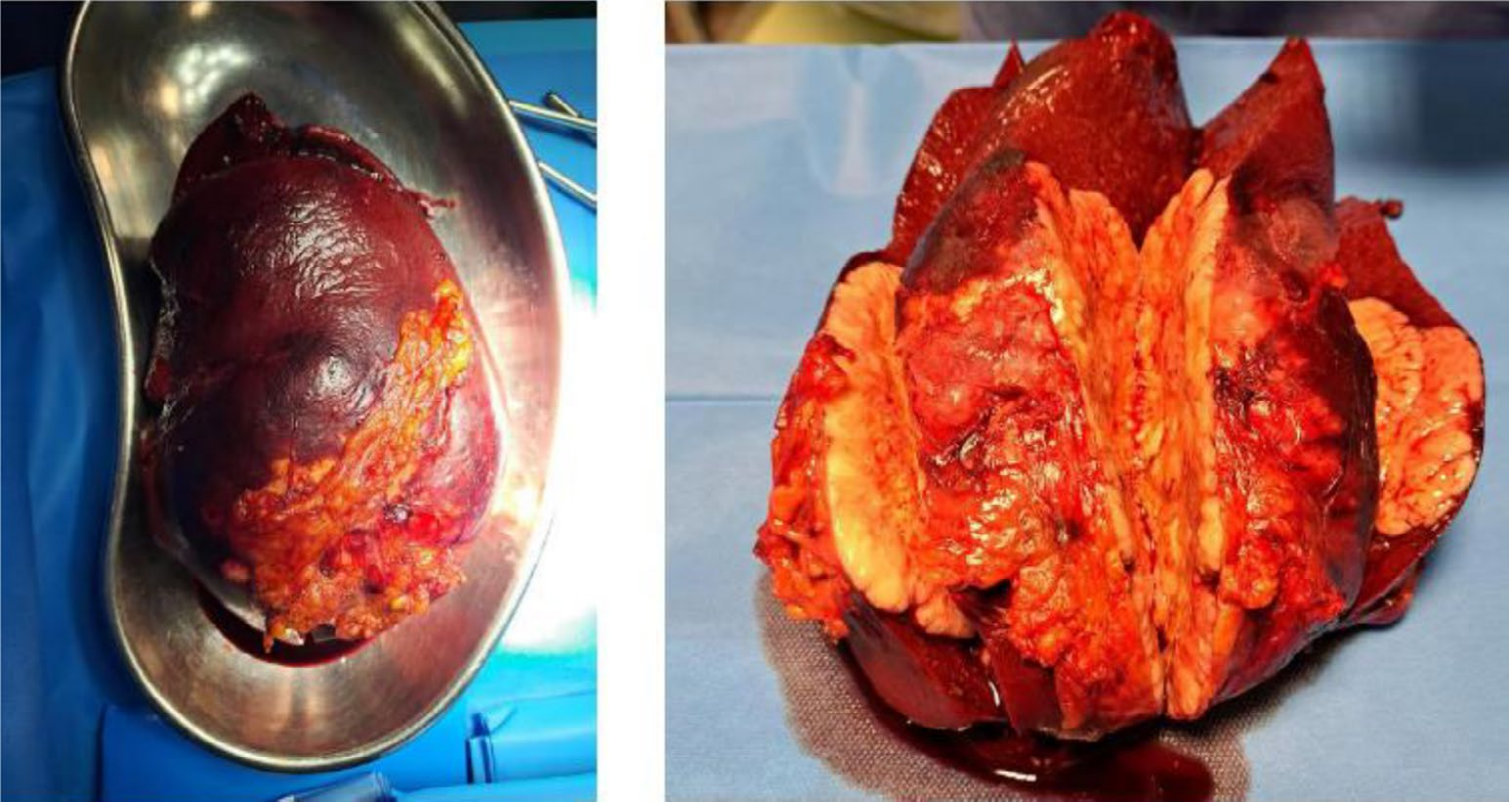
Splenectomy.

**FIGURE 4:**
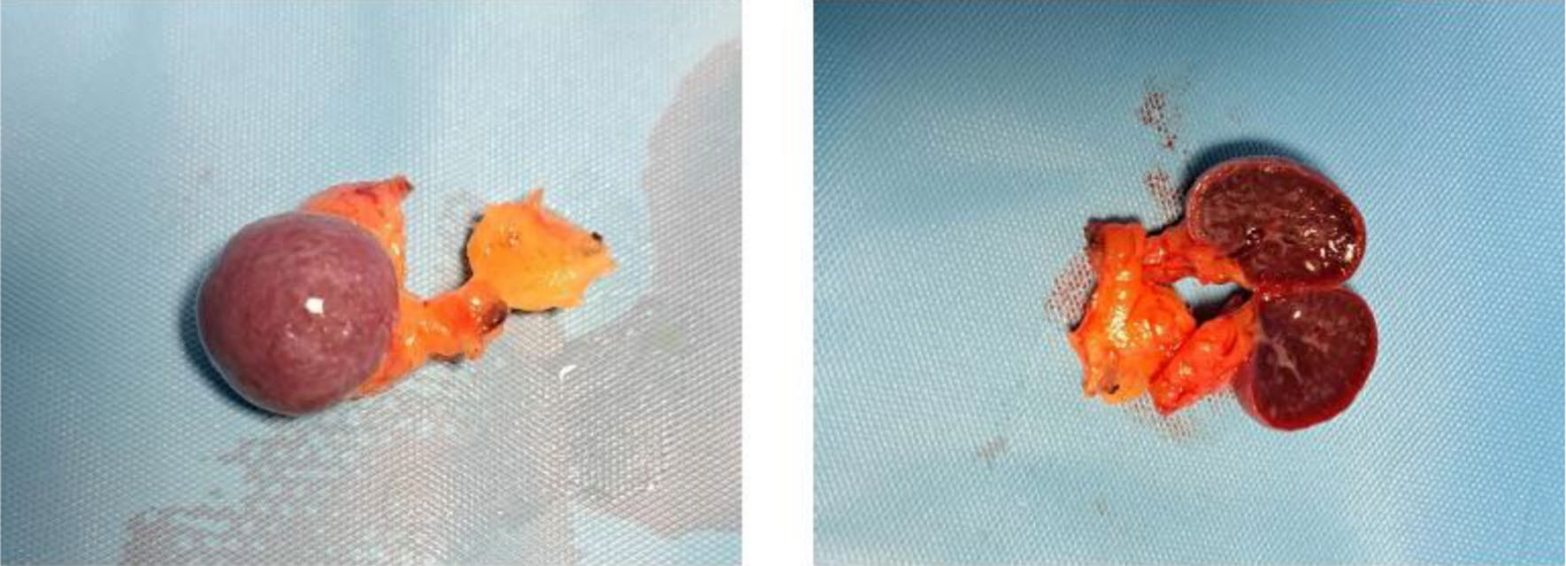
Accessory Spleen.

The surgical procedure included a diagnostic splenectomy and a wedge‐shaped biopsy of the third hepatic segment (Figure 3). The AS was also removed (Figure 4). Histological results confirmed the presence of diffuse large B‐cell lymphoma in both the spleen and the splenic hilum nodes. The accessory spleen and liver biopsy were negative, consistent with the preoperative FDG‐PET‐CT scan findings. Perioperative and postoperative antibiotics were administered, including Cefazolin (3 g/day for 5 days). Enoxaparin was administered postoperatively (4000 IU/day for 28 days).

The open splenectomy was performed under general balanced anesthesia with the patient in a supine position. A midline laparotomy was executed, and the spleen was removed, preserving parenchymal integrity.

The procedure was conducted by the lead surgeon from the hematology department.

No complications or adverse events were observed post‐surgery (Clavien‐Dindo Classification = 0). The surgery lasted 120 min, with a blood loss of 70 cc. No perioperative blood transfusions were required.

The patient was discharged on the 5th postoperative day. Within 15 days after surgery, she received vaccinations against encapsulated bacteria.

## 8 Follow Up and Outcomes

The hematological follow‐up with serial clinical and radiological evaluations lasted 29 months: no OPSI and disease recurrence were observed.

## 9 Discussion

Aside from the limitations of a case report, the strength of this paper lies in addressing the lack of specific guidelines regarding the appropriate surgical approach—whether to preserve or remove the accessory spleen—and in the limited number of similar surgical and medical cases reported in the literature. As a result, it remains uncertain whether sparing an accessory spleen after splenectomy is sufficient to mitigate asplenia and enhance immunological competence.

Splenectomy is occasionally necessary for patients with lymphoproliferative disorders (LPD). In these cases, splenectomy may be indicated for both therapeutic and diagnostic purposes. Therapeutic splenectomies (TS) are proposed for patients with unresponsive large neoplastic splenomegaly and poor quality of life [7, 8, 9]. Diagnostic splenectomy (DS) is typically recommended when peripheral localizations are absent or when lymph node excisional biopsy and/or bone marrow biopsy yield inconclusive results, as in our patient. Fine needle aspiration (FNA) and core needle biopsy (CNB) are alternative procedures to DS. However, FNA often fails to provide a definitive diagnosis in lymphoma cases, making CNB the preferred method. Nevertheless, CNB carries a complication rate of 8.2%–16.7%, with major complications occurring in 1%–1.9% of cases and minor complications in 5%–14.7% of cases [10, 11, 12, 13, 14, 15, 16]. In our case, splenectomy was scheduled as a diagnostic procedure because of the predominant splenic localization identified by preoperative FDG‐PET‐CT scan. Given the concordance between the FDG‐PET‐CT scan and histological findings regarding the accessory spleen (AS)—with no FDG uptake and no histological involvement—we questioned whether it was appropriate to remove the AS as a potential site of disease recurrence or to spare it in order to avoid asplenia. For patients with non‐neoplastic hematologic diseases, such as Immune Thrombocytopenic Purpura (ITP) [17, 18] or Hereditary Spherocytosis (HS) [19, 20, 21], the presence of an AS must be carefully evaluated and always removed because of the risk of therapy failure and disease recurrence. However, for patients undergoing splenectomy for suspected lymphoproliferative disorders (LPD), there are no established criteria or guidelines for deciding whether to remove or spare the AS. After conducting a literature review, we found no specific guidelines and only a few partially related case reports. As widely reported, asplenia following splenectomy exposes patients to complications and morbidities, particularly infections and sepsis, such as overwhelming post‐splenectomy infection (OPSI) syndrome [22, 23, 24, 25, 26, 27]. Therefore, vaccination protocols are strongly recommended [28, 29, 30, 31, 32]. It remains unclear whether sparing the accessory spleen (AS) prevents OPSI. In fact, some cases of OPSI have been reported in patients with an AS, though vaccination status was unknown [33]. Consequently, it is unclear if sparing the AS protects against OPSI and avoids the need for vaccination protocols. Regarding the impact of splenectomy on immune response capacity (IRC), it has been observed that the percentage of circulating IgM memory B cells is reduced in patients who underwent splenectomy. Measuring IgM memory B cells in the blood is a useful tool for assessing splenic function. Higher serum levels of IgM and IgG have been reported in patients with an AS compared to those who have had a splenectomy without residual AS [34]. This suggests that sparing the AS may help preserve IRC. We hypothesize that sparing the AS together with a proper post‐splenectomy vaccination schedule can improve the patient's immune competence. However, there are no conclusive data in the literature regarding the prognostic impact of sparing an AS if it is involved by lymphoproliferative disease (LPD).

We suggest that a negative FDG‐PET‐CT scan may reliably exclude involvement of the accessory spleen (AS), making it feasible to spare the AS during splenectomy. Conversely, if the AS is FDG‐PET‐CT scan positive, especially with high FDG uptake, it should be considered pathological and removed. In our case, the AS was both FDG‐PET‐CT scan‐negative and histologically uninvolved. However, Pitini et al. [35] reported a case where an AS was positive on FDG‐PET‐CT scan but histologically uninvolved in a patient with sclero‐nodular Hodgkin Lymphoma. Thus, according to current literature, it is not possible to definitively determine if patients with lymphoproliferative disorders (LPD) who are candidates for splenectomy should routinely have their AS removed, despite the procedure being relatively simple.

**FIGURE 5:**
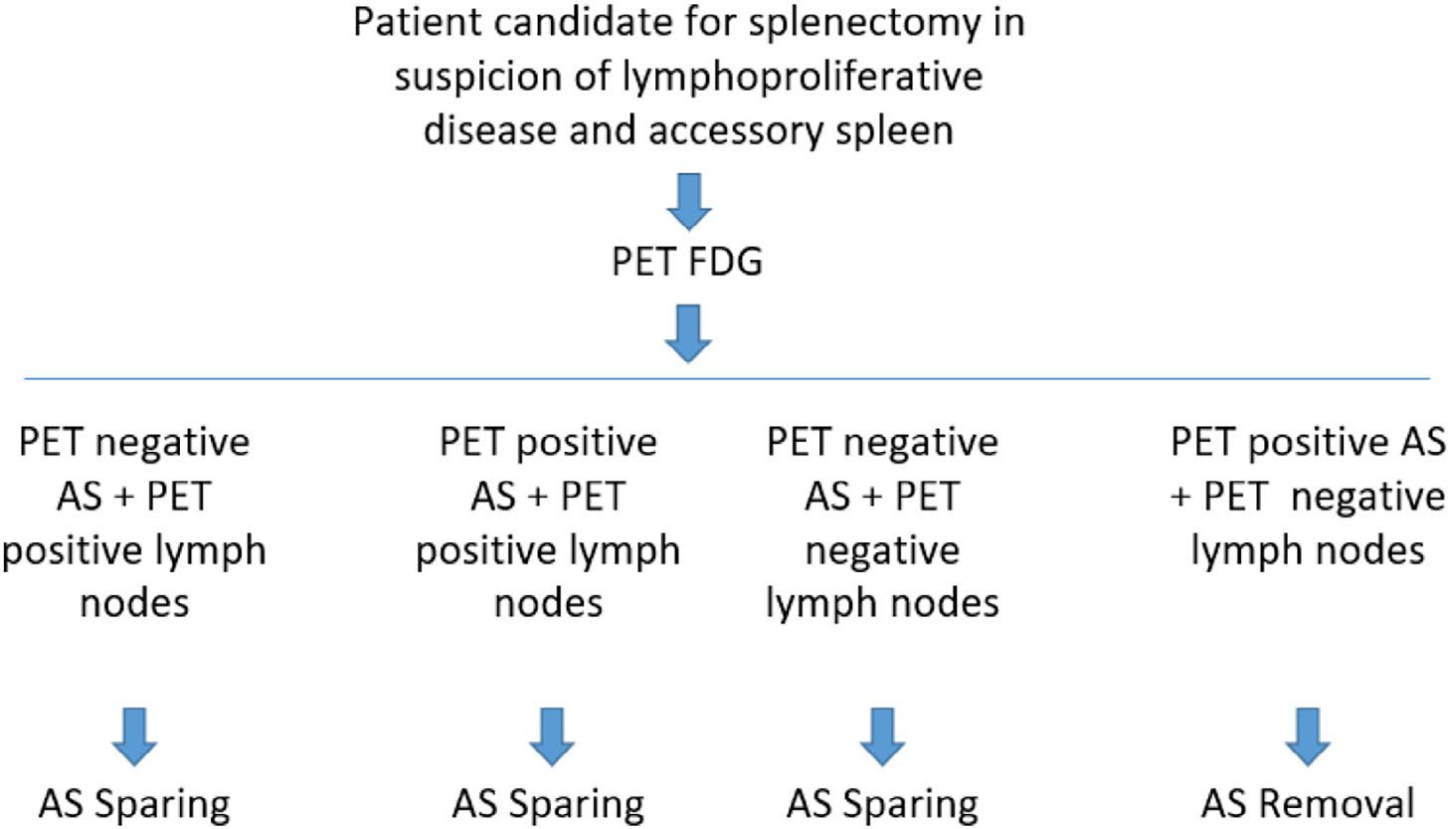
Therapeutic Algorithm.

Starting from the concept that every patient affected by DLBCL should be treated with chemotherapy after diagnostic splenectomy [36], we would like to propose a pivotal algorithm. In patients with an FDG‐PET‐CT scan negative AS, with or without other suspected disease localizations beyond the spleen, sparing the AS could be considered to preserve immune response capacity (IRC). On the other hand, in patients with an AS positive on FDG‐PET‐CT scan as the unique disease localization besides the spleen, removal of the AS may be indicated because of the risk of disease recurrence. For patients with an AS that is FDG‐PET‐CT scan‐positive but with additional lymphoproliferative disease (LPD) localizations, such as FDG‐PET‐CT scan‐positive abdominal lymph nodes or liver or bone marrow, sparing the AS may not have a negative prognostic impact (Figure 5), leaving the treatment of residual disease to post‐operative chemotherapy (R‐CHOP scheme).

Further retrospective or prospective studies are needed to evaluate the outcomes of sparing versus removing the AS. It is crucial to understand the implications of AS sparing on the patient's immunological function, risks of OPSI, and potential impact on disease recurrence or prognosis. Finally, it remains unclear whether to remove or preserve the AS in cases of splenectomy for diagnostic purposes when LPD is suspected, and the prognostic implications (both oncological and infectious) of these strategies are uncertain. Preoperative FDG‐PET‐CT scan findings regarding disease spread could likely guide the surgical decision.

## Author Contributions

**Valerio Rinaldi:** data curation, investigation, resources, writing – original draft. **Michela Ribersani:** methodology. **Carla Giordano:** validation. **Francesca Maccioni:** formal analysis. **Iulia Catalina Ferent:** software. **Priscilla Nardi:** software. **Roberto Caronna:** conceptualization, supervision, writing – review and editing. **Paolina Saullo:** conceptualization.

## Ethics Statement

Ethical review and approval were waived for this study because of its retrospective nature.

## Consent

Patient's written consent to data management and to publish this paper was obtained.

## Data Availability

Data sharing is not applicable to this article, as no datasets were generated or analyzed during the current study.

## References

[1] S. A. Bajwa and A. Kasi , Anatomy, Abdomen and Pelvis, Accessory Spleen (StatPearls, 2020). 30085582

[2] I. Varga , J. Babala , and D. Kachlik , “Anatomic Variations of the Spleen: Current State of Terminology, Classification, and Embryological Background,” Surgical and Radiologic Anatomy 40, no. 1 (2018): 21–29, 10.1007/s00276-017-1893-0. 28631052

[3] D. Ksiadzyna and A. S. Peña , “Abdominal Splenosis,” Revista Espanola de Enfermedades Digestivas 103, no. 8 (2011): 421–426, 10.53347/rid-96872. 21867352

[4] S. Mohammadi , A. Hedjazi , M. Sajjadian , N. Ghrobi , M. D. Moghadam , and M. Mohammadi , “Accessory Spleen in the Splenic Hilum: A Cadaveric Study With Clinical Significance,” Medical Archives 70, no. 5 (2016): 389–391, 10.5455/medarh.2016.70.389-391. 27994303 PMC5136441

[5] F. Lancellotti , L. Sacco , S. Cerasari , et al., “Intrapancreatic Accessory Spleen False Positive to 68Ga‐Dotatoc: Case Report and Literature Review,” World Journal of Surgical Oncology 17, no. 1 (2019): 117, 10.1186/s12957-019-1660-2. 31288823 PMC6617599

[6] L. L. Yavorkovsky , “Diffuse Large B Cell Lymphoma: Splenectomy as a Cure for a Spleen‐Limited Disease,” Leukemia & Lymphoma 61, no. 12 (2020): 3006–3009, 10.1080/10428194.2020.1788018. 32643483

[7] R. A. Agha , A. J. Fowler , A. Saeta , et al., “The SCARE Statement: Consensus‐Based Surgical Case Report Guidelines,” International Journal of Surgery 34 (2016): 180–186, 10.1016/j.ijsu.2016.08.014. 27613565

[8] O. Bairey , L. Shvidel , C. Perry , et al., “Characteristics of Primary Splenic Diffuse Large B‐Cell Lymphoma and Role of Splenectomy in Improving Survival,” Cancer 121, no. 17 (2015): 2909–2916, 10.1002/cncr.29487. 26096161

[9] N. Iriyama , A. Horikoshi , Y. Hatta , Y. Kobayashi , S. Sawada , and J. Takeuchi , “Localized, Splenic, Diffuse Large B‐Cell Lymphoma Presenting With Hypersplenism: Risk and Benefit of Splenectomy,” Internal Medicine 49, no. 11 (2010): 1027–1030, 10.2169/internalmedicine.49.3228. 20519821

[10] S. Barone , M. R. Baer , S. N. J. Sait , D. Lawrence , A. W. Block , and M. Wetzler , “Ultrasound‐Guided Fine Needle Biopsy of the Spleen: High Clinical Efficacy and Low Risk in a Multicenter Italian Study,” American Journal of Hematology 67, no. 2 (2001): 93–99, 10.1002/ajh.1085. 11343380

[11] M. Gómez‐Rubio , A. López‐Cano , P. Rendón , et al., “Safety and Diagnostic Accuracy of Percutaneous Ultrasound‐Guided Biopsy of the Spleen: A Multicenter Study,” Journal of Clinical Ultrasound 37, no. 8 (2009): 445–450, 10.1002/jcu.20608. 19582827

[12] S. Lieberman , E. Libson , B. Maly , P. Lebensart , D. Ben‐Yehuda , and A. I. Bloom , “Imaging‐Guided Percutaneous Splenic Biopsy Using a 20‐ or 22‐Gauge Cutting‐Edge Core Biopsy Needle for the Diagnosis of Malignant Lymphoma,” American Journal of Roentgenology 181, no. 4 (2003): 1025–1027, 10.2214/ajr.181.4.1811025. 14500223

[13] S. Lieberman , E. Libson , T. Sella , P. Lebensart , and J. Sosna , “Percutaneous Image‐Guided Splenic Procedures: Update on Indications, Technique, Complications, and Outcomes,” Seminars in Ultrasound, CT and MRI 28, no. 1 (2007): 57–63, 10.1053/j.sult.2006.10.001. 17366709

[14] M. C. Olson , T. D. Atwell , W. S. Harmsen , et al., “Safety and Accuracy of Percutaneous Image‐Guided Core Biopsy of the Spleen,” American Journal of Roentgenology 206, no. 3 (2016): 655–659, 10.2214/AJR.15.15125. 26901024

[15] V. F. I. Sangiorgio , H. Rizvi , J. Padayatty , et al., “Radiologically Guided Percutaneous Core Needle Biopsy of the Spleen: A Reliable and Safe Diagnostic Procedure for Neoplastic and Reactive Conditions,” Histopathology 78, no. 7 (2021): 1051–1055, 10.1111/his.14327. 33393079

[16] A. Tam , S. Krishnamurthy , E. P. Pillsbury , et al., “Percutaneous Image‐Guided Splenic Biopsy in the Oncology Patient: An Audit of 156 Consecutive Cases,” Journal of Vascular and Interventional Radiology 19, no. 1 (2008): 80–87, 10.1016/j.jvir.2007.08.025. 18192471

[17] K. L. Junus , J. A. Friedman , R. R. Rubin , B. A. Bianco , and A. E. Trebelev , “Accessory Spleen Embolization: An Option for Refractory Idiopathic Thrombocytopenic Purpura (ITP),” Diagnostic and Interventional Imaging 101, no. 2 (2020): 117–118, 10.1016/j.diii.2019.06.002. 31262671

[18] T. Worrest , E. Dewey , and L. E. Fischer , “Response Regarding: Missing Accessory Splenectomy as a Preventable Cause of Immune Thrombocytopenic Purpura Relapse,” Journal of Surgical Research 258 (2021): 463–464, 10.1016/j.jss.2020.10.001. 33160634

[19] J. B. Bart and M. F. Appel , “Recurrent Hemolytic Anemia Secondary to Accessory Spleens,” Southern Medical Journal 71, no. 5 (1978): 608–609, 10.1097/00007611-197805000-00038. 644372

[20] R. D. Croom , C. W. McMillan , E. P. Orringer , and G. F. Sheldon , “Hereditary Spherocytosis: Recent Experience and Current Concepts of Pathophysiology,” Annals of Surgery 203, no. 1 (1986): 34–39, 10.1097/00000658-198601000-00007. 3942420 PMC1251036

[21] S. I. Schwartz , “Role of Splenectomy in Hematologic Disorders,” World Journal of Surgery 20, no. 9 (1996): 1156–1159, 10.1007/s002689900176. 8864075

[22] A. Di Sabatino , R. Carsetti , and G. R. Corazza , “Post‐Splenectomy and Hyposplenic States,” Lancet 378, no. 9785 (2011): 86–97, 10.1016/S0140-6736(10)61493-6. 21474172

[23] D. H. Morris and F. D. Bullock , “The Importance of the Spleen in Resistance to Infection,” Annals of Surgery 70, no. 5 (1919): 513–531, 10.1097/00000658-191911000-00001. 17864185 PMC1410445

[24] N. Bisharat , H. Omari , I. Lavi , and R. Raz , “Risk of Infection and Death Among Post‐Splenectomy Patients,” Journal of Infection 43, no. 3 (2001): 182–186, 10.1053/jinf.2001.0904. 11798256

[25] H. King and H. B. Shumacker , “Splenic Studies. I. Susceptibility to Infection After Splenectomy Performed in Infancy,” Annals of Surgery 136, no. 2 (1952): 239–242, 10.1097/00000658-195208000-00006. 14953147 PMC1802258

[26] F. Tahir , J. Ahmed , and F. Malik , “Post‐Splenectomy Sepsis: A Review of the Literature,” Cureus 12 (2020): e6898, 10.7759/cureus.6898. 32195065 PMC7059871

[27] C. Theilacker , K. Ludewig , A. Serr , et al., “Overwhelming Postsplenectomy Infection: A Prospective Multicenter Cohort Study,” Clinical Infectious Diseases 62, no. 7 (2016): 871–878, 10.1093/cid/civ1195. 26703862

[28] P. Bonanni , M. Grazzini , G. Niccolai , et al., “Recommended Vaccinations for Asplenic and Hyposplenic Adult Patients,” Human Vaccines and Immunotherapeutics 13 (2017): 359–368, 10.1080/21645515.2017.1264797. 27929751 PMC5328222

[29] F. Casciani , M. T. Trudeau , and C. M. Vollmer , “Perioperative Immunization for Splenectomy and the Surgeon's Responsibility,” JAMA Surgery 155, no. 11 (2020): 1068, 10.1001/jamasurg.2020.1463. 32936229

[30] J. M. Davies , M. P. N. Lewis , J. Wimperis , I. Rafi , S. Ladhani , and P. H. B. Bolton‐Maggs , “Review of Guidelines for the Prevention and Treatment of Infection in Patients With an Absent or Dysfunctional Spleen: Prepared on Behalf of the British Committee for Standards in Haematology by a Working Party of the Haemato‐Oncology Task Force,” British Journal of Haematology 155, no. 3 (2011): 308–317, 10.1111/j.1365-2141.2011.08843.x. 21988145

[31] N. Murthy , A. P. Wodi , S. Cineas , et al., “Recommended Adult Immunization Schedule, United States, 2023,” Annals of Internal Medicine 176, no. 3 (2023): 367–380, 10.7326/M23-0041. 36757885

[32] “Piano Nazionale di Prevenzione Vaccinale (PNPV) 2023–2025,” (2023), https://www.epicentro.iss.it/vaccini/piano‐nazionale‐vaccini‐2023‐2025.

[33] N. T. Connell , A. M. Brunner , C. A. Kerr , and F. J. Schiffman , “Splenosis and Sepsis: The Born‐Again Spleen Provides Poor Protection,” Virulence 2, no. 1 (2011): 4–11, 10.4161/viru.2.1.14611. 21224728

[34] R. Leemans , W. Manson , J. A. M. Snijder , et al., “Immune Response Capacity After Human Splenic Autotransplantation,” Annals of Surgery 229, no. 2 (1999): 279–285, 10.1097/00000658-199902000-00017. 10024111 PMC1191642

[35] V. Pitini , G. Navarra , V. Cavallari , and C. Arrigo , “An Accessory Spleen Wrongly Recognised as Relapse by Positron Emission Tomography,” European Journal of Haematology 77, no. 3 (2006): 270–271, 10.1111/j.1600-0609.2006.00690.x. 16923117

[36] C. C. H. M. Maas , D. van Klaveren , M. Durmaz , et al., “Comparative Effectiveness of 6x R‐CHOP21 Versus 6x R‐CHOP21 + 2 R for Patients With Advanced‐Stage Diffuse Large B‐Cell Lymphoma,” Blood Cancer Journal 14, no. 1 (2024): 157, 10.1038/s41408-024-01137-0. 39266543 PMC11393348

